# Intussusception hospitalizations incidence in the pediatric population in Italy: a nationwide cross-sectional study

**DOI:** 10.1186/s13052-016-0298-8

**Published:** 2016-09-27

**Authors:** Francesco Trotta, Roberto Da Cas, Antonino Bella, Carmela Santuccio, Stefania Salmaso

**Affiliations:** 1 Pharmacovigilance Office, Italian Medicines Agency (AIFA), Via del Tritone 181, Rome, 00187 Italy; 2 National Centre for Epidemiology, Surveillance and Health Promotion, Italian National Institute of Health, Viale Regina Elena 299, Rome, 00161 Italy

**Keywords:** Intussusception, Incidence, Rotavirus vaccine, Vaccine surveillance, Children

## Abstract

**Background:**

Study to investigate the intussusception incidence background in the pediatric population and its temporal trend in Italy.

**Methods:**

A cross-sectional study was conducted on the pediatric population aged 0 to 15 years, in the period 1 January 2002 to 31 December 2012. Intussusception cases were identified using the national hospital discharge database. The annual intussusception incidence, the incidence rate ratios (IRRs) and the related 95 % confidence Intervals (CI) were calculated.

**Results:**

The overall intussusception incidence rate was 21 per 100,000 children aged ≤15 years, and was higher among boys than girls. The highest intussusception incidence rate occurred in infants <1 year of age (39 per 100,000 infants). Among infants, incidence varied with the geographical area, with higher rates in the central Italy (50 per 100,000 infants).

The annual incidence rates in infants were stable since 2004 and up to 2012, ranging from 40.1 and 33.0 per 100,000 infants. Similar stable patterns were observed when conducting the analysis on children over 1 year of age.

**Conclusions:**

This study provided the intussusception incidence background in Italy in different pediatric ages, including infants, over an 11-year period. This information is essential in post-marketing safety surveillance, to continuously monitor the benefit/risk profile of rotavirus vaccinations.

**Electronic supplementary material:**

The online version of this article (doi:10.1186/s13052-016-0298-8) contains supplementary material, which is available to authorized users.

## Background

Intussusception is the most common cause of acute intestinal obstruction in children under 2 years of age [1]. Less than 5 % of cases resolve spontaneously and if treated early, almost all cases can be reduced by enema or surgery [2].

The etiology of intussusception is unknown for most of the cases, although some conditions have been found associated [3–8].

Rotashield®, the first-generation rotavirus vaccine (RV) was found to be associated with intussusception in infants, leading to its withdrawal from the market in 1999 [8]. In Italy two different RVs (Rotarix® and Rotateq®) have been available on the market since 2007, being administered as a two-or three-dose schedule starting from 6 weeks of age [9, 10]. Neither of the two licensed RV were found to be associated with intussusception in clinical trials [11, 12]. However, post-marketing studies enrolling much larger population have suggested an intussusception risk following RV vaccination, which is in 2 to 5 excess cases per 100,000 vaccinated infants [13–16].

Although vaccination against rotavirus is not included in the universal National Immunization Program (2012–2014), some Italian regions introduced the RV targeting specific children subgroups and/or with different reimbursement schemes [17]. According to official data in Italy during 2013, approximately 76,000 vaccine doses were purchased within the Italian national health system (NHS), resulting in almost 37,000 vaccinated children (13,700 doses were purchased in 2010 by the NHS) [18, 19]. This led the *Italian Pharmacovigilance Network* to capture the first spontaneous intussusception report in 2012 [18].

According to literature, the EU intussusception incidence background in the pediatric population ranges between 0.66 and 2.24 per 1000 children admitted to hospital, and between 0.75 and 1.00 per 1000 children admitted to the emergency ward [20]. This estimate was largely based on hospital discharge data collected before 1995. A more recent review estimated the intussusception incidence background in infants (aged ≤1 year) ranging between 20 and 66 per 100,000 infants [21]. The reported incidence in different countries varies largely, possibly due to different factors (age, patient settings and pathological conditions, socioeconomic status, and geographical area) [20, 21]. Only one incidence study was conducted in Italy in a primary care pediatric setting (children aged <10 years) and provided an estimate of 5.0 per 100,000 person-years [22]. However, it is well known that intussusceptions cases are generally treated in a hospital setting [21].

The use of RV is expected to increase in Italy, thus any post-marketing surveillance would require intussusception incidence background data from reliable and stable sources.

Therefore, a cross-sectional study was conducted based on the national database of hospital discharges in the period 1 January 2002 to 31 December 2012. The study was aimed to determine the overall intussusception incidence in the pediatric population in Italy, and to describe its temporal trends over a period of time in specific age groups. The study included the evaluation of incidence patterns according to intussusception severity, gender, geographical area, and pathological lead points. The potential changes of intussusception rates in different timeframes was also explored in infants, before and after 2007 (before and after the marketing authorization of RV in Italy).

## Methods

### Population and study settings

The study was conducted on the Italian pediatric population aged 0 to 15 years; cases were identified from hospitalization records. Only children with an intussusception discharge diagnosis in the study period were included. Two different sub-cohorts were identified for the main analyses, i.e. infants aged 0 to < 1 years and children aged ≥ 1 year of age.

### Data sources

In Italy the NHS is provided universally free. A hospital database collects all hospital discharge records (including day-hospital/day-surgery admission). Only routinely collected information was used in the study and data were analyzed through a unique, anonymised, personal identifier. The following data were extracted for the present analyses from each record: age, gender, region of residence, admission and discharge dates, diagnosis (primary and secondary) and procedures [coded according to the International Classification of Diseases (ICD), 9^th^Revision],status at discharge (deceased/non-deceased).

### Case definition

Records with the following intussusceptionICD-9 codes, either as primary or secondary diagnosis, were selected from the national database hospital discharge records: 560.0 (intussusception) and 543.9 (other and unspecified appendix diseases). The analysis was restricted to incident cases identified from 1 January 2002 to 31 December 2012; thus, only the first intussusception-associated hospitalizations (considered as index dates) were included.

### Identification of risk factors potentially associated with intussusception

To identify potential risk factors for intussusception, all hospitalization records in the 6-month period preceding the index date (the first intussusception diagnosis) for each identified case were retrieved. Hospitalization records in 2001 were used to collect the medical history of intussusception case incidents in 2002. A predefined list for the leading points pathology was used with the related ICD-9 codes (see Additional file 1: Annex 1).

### Case Severity

As a proxy for the case severity, ICD-9 procedure codes were used to identify those intussusception hospitalizations requiring surgical or radiological intervention. Surgical intervention was defined by procedure codes 46.80 to 46.82, and radiological intervention by codes 96.29 and 96.39. All the procedure codes were coupled to an intussusception code, namely in the same hospital discharge form of the intussusception incident case. Three categories were defined: i) intussusception (identified by diagnosis); ii) intussusception requiring surgery (identified by diagnosis and surgical procedures); iii) intussusception requiring non-surgical intervention (identified by diagnosis and non-surgical procedures). The in-hospital mortality was also evaluated (number of children who died in-hospital following the first intussusception episode).

### Intussusception Recurrence

Consecutive intussusception hospitalizations for the same child were identified for up to 1 year following the first episode of intussusception (incident case). The same ICD-9 codes applied to identify incident cases were used to retrieve recurrence of intussusception episodes. Three categories of recurrence were defined: i) early recurrence: cases with hospital readmission ≤7 days from the first episode; ii) medium term recurrence: cases with hospital readmission between 8 and 30 days from the discharge; iii) late recurrence: cases with hospital readmission after 30 days from the discharge and ≤1 year.

### Statistical analysis

The annual intussusception incidence per 100,000 infants/children was calculated using as a reference the Italian population resident figure provided by the *National Institute for Statistics,* for the period 2002 to 2012 [23]. Births were assumed to be evenly distributed throughout the year, when hospitalization rates by age (in months) were calculated. Intussusception period specific incidence rates associated with hospitalization were estimated. Annual incidence rates were calculated after being adjusted by age and gender, through direct standardization methods, using the 2012 population as a reference to take into account the ongoing demographic variation.

The incidence rate ratios (IRRs), and the 95 % Confidence Intervals (CI) were calculated through the Poisson regression. Statistical analyses were performed using STATA software (version 11.2; Stata Corp LP, College Station, TX, USA) and IBM SPSS Statistics for Windows (Version 22.0; Armonk, NY: IBM Corp.).

## Results

**Fig. 1:**
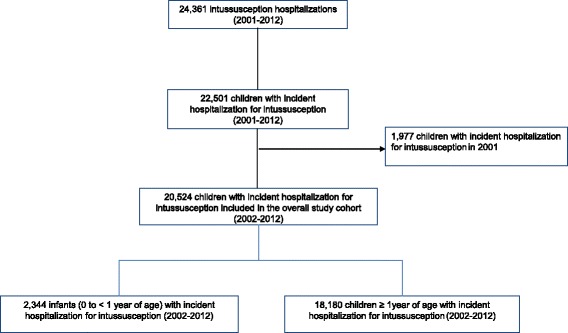
Flowchart of the enrolled cohort of children with incident hospitalization for intussusception

Overall, 20,524 children aged 0 to 15 years were identified as intussusception incident cases during the 2002 to 2012 timeframe, 2344 were infants aged 0 to < 1 year (Fig. 1).

### Intussusception incidence rates by age, gender, geographical area, severity and risk factors

The description of the characteristics of the overall pediatric population enrolled is shown in Table 1. The intussusception incidence rate was 21 per 100,000 children and was higher among boys than girls (23 per 100,000 vs 19 per 100,000, respectively); thus boys had a statistically significant increased probability to experience intussusception hospitalization (IRR: 1.16; 95 % CI 1.13–1.20). Rates of intussusceptions also varied substantially by age, with the highest incidence rate occurring in infants <1 year of age (39 per 100,000 infants). However, rates were low for infants <14 weeks (19 per 100,000 infants in the group aged 6 to 14 weeks), then increased rapidly, peaking at 60 per 100,000 for infants aged 25 to 32 weeks, then decreasing for children ≤6 years of age; a rise in the hospitalization incidence rates occurred also within age groups 10 to 12 years (26 per 100,000 children). The probability of intussusception hospitalization became statistically significant higher from 15 weeks of age to ≤52 weeks (the risk was quadrupled for infants aged 25 to 32 weeks, IRR: 3.94, 95 % CI 3.15–4.93.). The incidence rates measured in the overall pediatric population varied by geographical areas, showing a marked increase in rates in the south and central Italy, with respect to the north of Italy (IRR south: 2.65; 95 % CI 2.56–2.74; IRR central: 1.61; 95 % CI 1.54–1.68).

**Table 1:** Characteristics of intussusception incident cases included in the overall study cohort (2002–2012)

	No.	%	Incidence (per 100,000)	IRR (IC 95 %)
Total	20,524		21	
Male	11,321	55.2	23	1.16 (1.13–1.20)
Female	9203	44.8	19	Ref
Age				
1–5 weeks	89	0.4	15	Ref
6–14 weeks	196	1.0	19	1.22 (0.95–1.57)
15–24 weeks	541	2.6	47	3.04 (2.43–3.80)
25–32 weeks	561	2.7	60	3.94 (3.15–4.93)
33–52 weeks	957	4.7	41	2.69 (2.16–3.34)
1–3 years	2794	13.6	15	1.00 (0.81–1.23)
4–6 years	2699	13.2	15	0.97 (0.79–1.20)
7–9 years	4349	21.2	24	1.56 (1.27–1.93)
10–12 years	4875	23.8	26	1.72 (1.40–2.21)
13–15 years	3463	16.9	18	1.20 (0.97–1.48)
Geographical area				
North Italy	4968	24.2	12	Ref
Central Italy	3408	16.6	19	1.61 (1.54–1.68)
South Italy, Sicily and Sardinia	12,148	59.2	32	2.65 (2.56–2.74)
Risk factors (previous 6-month hospitalizations)				
Yes	291	1.4		
No	20,233	98.6		
Severity				
Intussusception	18,000	87.7		
Intussusception requiring surgery	2036	9.9		
Intussusception requiring enema	488	2.4		
Recurrence (1 year following the first episode)				
Yes	759	3.7		
No	19,765	96.3		

In the vast majority of cases (87.7 %) non-surgical, or -radiological procedures were reported, shown in Table 1.

Overall, 8 children hospitalized for intussusception died in-hospital (3 in the first year of life), with an in-hospital mortality rate of 0.39 per 1000, in the study population. The incidence rate of in-hospital mortality following intussusception in infants (<1 year of age) was 0.5 per million infants (Additional file 1: Figure S1).

About 4 % of the children hospitalized with an intussusception diagnosis had a recurrence within 1 year after the first episode (Table 1). The 41.6 % of children experiencing a recurrence were hospitalized ≤30 days from the intussusception incident episode (Additional file 1: Figure S2).

Only 291 (1.4 %) children showed at least one known intussusception risk factor in the 6-month period prior to the incident episode (Table 2). The most frequent risk factors were presented in Table 2 and included gastroenteritis (44.3 % of the cases) followed by inflamed appendix, and Henoch-Schonlein purpura (35.1 % and 9.6 %, respectively).

**Table 2:** Risk factors distribution among intussusception incident cases included in the overall study cohort (2002–2012)

	No.	%
Total (at least one risk factor)	291	
Inflamed appendix	102	35.1
Gastroenteritis	129	44.3
Cystic fibrosis	5	1.7
Hirschsprung disease	2	0.7
Reduplication/intestinal duplication	1	0.3
Lymphoma	3	1.0
Intestinal tumor	6	2.1
Henoch-Schönlein purpura	28	9.6
Meckel diverticulum	2	0.7
Intestinal polyps (benign tumor)	3	1.0
Peutz-Jeghers syndrome	4	1.4
Nephrotic syndrome	6	2.1
Kawasaki disease	2	0.7
Ectopic pancreas in ileum	1	0.3
Hemolytic-uremic syndrome	1	0.3

### Intussusception incidence rate temporal trends in infants and children aged at least 1 year

**Fig. 2:**
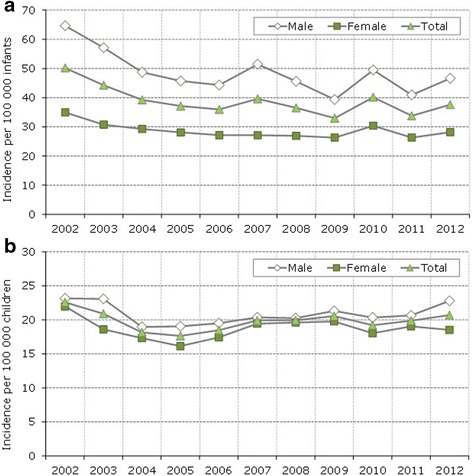
**a** Intussusception annual incidence rate (2002–2012) in infants <1 year of age standardized by age and gender (using the Italian resident population in 2012). **b** Intussusception annual incidence rate (2002–2012) in children 1–15 years of age standardized by age and gender (using the Italian resident population in 2012)

The annual incidence rate in children <1 year of age was highest in 2002 and decreased steadily to 2004 falling from 50.2 to 39.2 per 100,000 infants, rates then remained stable from 2005 to 2012, ranging from 33.0 to 40.1 per 100,000 infants (Fig. 2a). Stable temporal trends were observed when considering incidence rates by gender, with constantly higher incidence rates in boys in each year.

Similar temporal trends were observed when the analysis was conducted among children aged 1–15 years (Fig. 2b), although the incidence was lowest in this population (ranging from 17.6 to 22.6 per 100,000 children in the considered period).

**Fig. 3:**
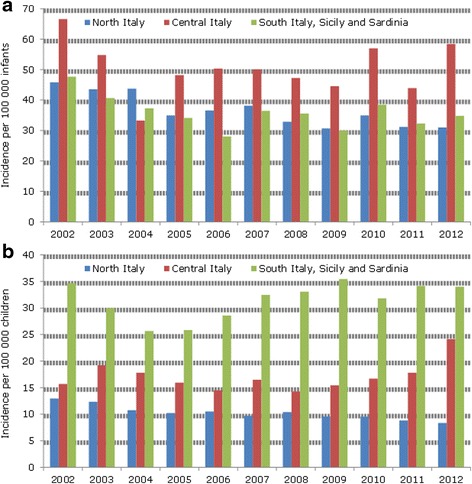
**a** Intussusception annual incidence rate (2002–2012) in infants <1 year of age by geographical area. **b** Intussusception annual incidence rate (2002–2012) in children 1–15 years of age by geographical area

The annual trends of incidence rates by geographical areas remained overall stable (Figs. 3a,b). Specifically, when considering the infants cohort, the highest incidence rates were observed in each year in the central Italy: decreasing from 66.6 per 100,000 infants in 2002 to 58.5 in 2012. In the central Italy, the highest incidence rate was observed in Tuscany and Umbria; region specific incident rates in infants were showed in Additional file 1: Figure S3a.

With regard to the pediatric cohort over 1 year of age, a constant higher incidence trend in the period 2002 to 2012 was observed in south of Italy which was 3 fold higher compared to the northern (Fig. 3b). The highest rates were observed in the Sicily, Puglia and Basilicata (Additional file 1: Figure S3b).

The attempt in comparing intussusception hospitalization rates in infants across different time intervals, before and after 2007 (before and after RV use in Italy), stratified by age in months did not show any increases in the first year of age during post-vaccine introduction years, when compared with pre-vaccine introduction rates (Additional file 1: Table S1). Overall, the IRR of intussusception was 0.92 in 2007–2008 (95 % CI: 0.83–1.03) and 0.89 in 2009–2010 (95 % CI: 0.79–1.00) and 0.87 in 2011–2012 (95 % CI: 0.77–0.97).

## Discussion

This is the first study providing the intussusception incidence background in Italy over an 11-year period in the whole pediatric population. The determined background rates in Italy in children <1 year of age (39 per 100,000 infants) is within the ranges reported at the EU level [21] and in line with those detected in Germany [24], Finland [25], UK and Ireland [26], and Switzerland [27], observed over comparable timeframes. The infant intussusception incidence measured in Italy is also closely similar to those detected at the US level [28–32].

Considering the entire pediatric population (aged ≤15 years) the incidence rates observed in Italy (21 per 100,000) is lower than those in other studies conducted in Norway [33], France [34] or Denmark [35]. However, it should be noted that all three studies in the mentioned countries were conducted in different timeframes, or with a limited enrolled population, which may explain the different intussusception incidence estimates when compared with those of Italy.

In the Italian pediatric population, incidence rates were found stable over time when considering different age groups. Few studies have reported details of temporal trends for intussusception. A Danish study reported a constant decrease in the incidence rates of infants ≤1 year from 1980 to 2001 [35]; this was explained by a possible shift in the management of intussusception from in-patient to short stay hospitalization in out-patient settings [36]. In some other literature the annual incidence rate of intussusception hospitalization was stable in the different calendar years [28, 37].

The higher incidence rate in boys was expected according to the already available data [20]. There was a strong variability in the rates for the Italian geographical regions (from 12/100,000 in the north to 32/100,000 in the south of Italy) that remained stable in the considered period. Moreover, geographical variability also appeared to be influenced by the age groups considered, being highest in the Centre in infants. Geographical and environmental variation in intussusception incidence is known [20, 21]; however, it should be pointed out that organizationally different clinical practices among the Italian regions may have contributed to varying rates. The only Italian study which was conducted in a primary care setting did not reveal such geographical differences, as the majority of family pediatricians included in the study were in the north of Italy [22].

The percentages of serious cases (9.9 %) observed in this study namely, those requiring surgical procedures are in line with data reported elsewhere in Europe [21]. However, the estimate of intussusception cases in Italy requiring enema appears to be too low (2.4 %) compared to those expected at EU level (77 %) [21]. The in-hospital intussusception mortality is a rare event in Italy (about 1 case per 2 million infants), and the rate is lower than that of the US study [38]. However, hospital-based data may underestimate the true mortality for intussusception since children dying before being hospitalized, or after being discharged were not taken into account.

In this study the overall intussusception recurrence rate of 3.7 % was consistent with previously published data [37, 39–41]. In our setting, 24.2 % of incident cases showed intussusception recurrences, ≤7 days from the first episode. The timing of recurrence was evaluated only in a few trials included in a recent meta-analysis on intussusception enema reduction, which showed a low recurrence rate at 48 h (≤5 %) [41].

The risk factors potentially linked to intussusception cases identified in this study were consistent with available data, where gastroenteritis and appendicitis were frequently recognized as pathological leading points [24, 27, 39–42].

The data in this study do not reveal any change in intussusception hospitalization rates among Italian infants (≤1 year of age) when comparing different time intervals before and after 2007 (before and after the marketing authorization of RV in Italy), although the coverage was very limited, <1 % of the birth cohort.

The main strength of this study is the large cohort of children enrolled. Italian birth cohorts were in fact included over an 11-year timeframe, thus these findings should be considered as representative of the whole country. Moreover, in Italy health care is provided free to the whole population within the NHS. For this reason, all intussusception hospitalizations were included in this study.

This study has several limitations. Intussusception hospitalization was determined on the basis of ICD-9 diagnosis code at hospital discharge without any prior validation of the diagnosis. Therefore, the Brighton Collaboration (BC) case definition on intussusception requiring specific clinical examinations as well as sign or symptoms not retrievable through hospital records, was not applicable in the context of our study [43]. However, a study conducted by Ducharme et al. in Canada, reported ICD-9 codes to be sensitive (89.3 %) and highly specific (>99.9 %) in identifying patients with intussusception from administrative data [44]. In addition, a US study conducted in 3 hospitals showed that almost 90 % of the intussusceptions codes collected from the discharge forms had met the highest level of diagnostic certainty [28]. Therefore, the mentioned details give support and negate the possibility of the misclassification of intussusception cases of this study.

Since the analysis included in this study is of only hospitalization data, the intussusception cases managed in an out-patient setting are not included, leading to a potential underestimation of the true incidence rate. However, in Italy pediatric patients with intussusception (especially infants) were directly admitted to hospital without any prior primary care referral [22].

## Conclusions

Although this study is based on routinely collected data, it still provides a robust and representative intussusception incidence background in Italy in the different age groups and evaluates its variability over an 11-year period. This knowledge is essential for post-marketing safety surveillance on rotavirus vaccinations and to provide information useful for vaccine-safety policies.

## Additional file

Additional file 1:
**Annex 1.** ICD-9 codes used in the identification of risk factors potentially associated to intussusception. **Figure S1.** In-hospital intussusception mortality incidence rate by age (2002–2012). **Figure S2.** Intussusception case incident distribution by recurrence time (1 year following the first episode) within the overall pediatric cohort. **Figure S3.** a Cumulative intussusception incidence rate among infants <1 year of age by region (2002–2012). **Figure S3.** b Cumulative intussusception incidence rate among children 1–15 years of age by region (2002–2012). **Table S1.** Intussusception hospitalization rate comparisons in different timeframes (before and after the marketing authorization RV vaccines in Italy) for infants aged <1 year. (DOCX 137 kb)
