# Supplementary material for: Intussusception hospitalizations incidence in the pediatric population in Italy: a nationwide cross-sectional study
Source: Ital J Pediatr. 2016 Sep 27;42:89. doi: 10.1186/s13052-016-0298-8 (PMC5039877; doi:10.1186/s13052-016-0298-8)
Supplement: Additional file 1: — Annex 1. ICD-9 codes used in the identification of risk factors potentially associated to intussusception. Figure S1. In-hospital intussusception mortality incidence rate by age (2002–2012). Figure S2. Intussusception case incident distribution by recurrence time (1 year following the first episode) within the overall pediatric cohort. Figure S3. a Cumulative intussusception incidence rate among infants <1 year of age by region (2002–2012). Figure S3. b Cumulative intussusception incidence rate among children 1–15 years of age by region (2002–2012). Table S1. Intussusception hospitalization rate comparisons in different timeframes (before and after the marketing authorization RV vaccines in Italy) for infants aged <1 year. (DOCX 137 kb) [file 13052_2016_298_MOESM1_ESM.docx]

**Supplementary Material**

**Annex 1. ICD-9 codes used in the identification of risk factors potentially associated to intussusception**

To identify intussusceptions potential risk factors, all identified cases of hospitalizations were collected in the 6-month period before the index date (intussusception first diagnosis). The following risk factors were considered as ‘known’ leading point pathologies:

- Meckel diverticulum (icd-9: 751.0; 152.3);
- Intestinal Polyps (benign tumor) (icd-9: 211.0-211.4);
- Peutz-Jeghers syndrome (icd-9: 759.6);
- [Henoch-Schönlein purpura](http://www.patient.co.uk/doctor/henoch-schonlein-purpura) (icd-9: 287.0);
- Lymphoma; lymph sarcoma; intestinal lymphoma; and lymphoid hyperplasia; (icd-9: 200.X; 201.X; 202.X);
- Intestinal tumor (150-159);
- Reduplication/intestinal duplication - a process by which the bowel wall is duplicated (icd-9: 751.8; 751.5);
- [Cystic fibrosis](http://www.patient.co.uk/doctor/cystic-fibrosis)(icd-9: 277.0);
- Inflamed appendix (icd-9: 540.x; 541; 542; 543.0);
- Ectopic pancreas in ileum (icd-9: 751.7; 157.8);
- Gastroenteritis (icd-9: 8.6; 8.8; 9.0-9.3);
- Hirschsprung disease (icd-9: 751.3);
- Mesenteric hernia (icd-9: 553.8; 553.9);
- Hemolytic-uremic syndrome (icd-9: 283.11);
- Idiopathic thrombocytopenic purpura (icd-9: 287.31);
- Nephrotic syndrome (icd-9: 581);
- Kawasaki disease (icd-9: 446.1);

**Supplementary figure 1. In-hospital intussusception mortality incidence rate by age (2002-2012)**

**Supplementary figure 2. Intussusception case incident distribution by recurrence time (1 year following the first episode) within the overall pediatric cohort.**

**Supplementary figure 3a. Cumulative intussusception incidence rate among infants <1 year of age by region (2002-2012).**

**Supplementary figure 3b. Cumulative intussusception incidence rate among children 1-15 years of age by region (2002-2012).**

**Supplementary table 1. Intussusception hospitalization rate comparisons in different timeframes (before and after the marketing authorization RV vaccines in Italy) for infants aged <1 year.**

|  | **2002-2006 Incidence* (ref)** | **2007-2008** | | **2009-2010** | | **2011-2012** | |
| --- | --- | --- | --- | --- | --- | --- | --- |
| **Age (months)** |  | **Incidence*** | **IRR (CI 95%)** | **Incidence*** | **IRR (CI 95%)** | **Incidence*** | **IRR (CI 95%)** |
| **1** | ***16*** | 22 | 1.31 (0.76-2.26) | 15 | 0.90 (0.49-1.67) | 17 | 1.01 (0.55-1.83) |
| **2** | ***13*** | 5 | 0.42 (0.16-1.08) | 12 | 0.90 (0.45-1.81) | 15 | 1.20 (0.63-2.27) |
| **3** | ***25*** | 17 | 0.68 (0.39-1.18) | 17 | 0.67 (0.38-1.16) | 13 | 0.52 (0.28-0.97) |
| **4** | ***39*** | 45 | 1.16 (0.80-1.67) | 34 | 0.87 (0.58-1.30) | 28 | 0.70 (0.45-1.10) |
| **5** | ***55*** | 39 | 0.71 (0.49-1.03) | 45 | 0.81 (0.57-1.15) | 52 | 0.95 (0.68-1.33) |
| **6** | ***55*** | 47 | 0.87 (0.61-1.22) | 50 | 0.91 (0.65-1.27) | 40 | 0.73 (0.50-1.05) |
| **7** | ***69*** | 63 | 0.90 (0.67-1.22) | 57 | 0.82 (0.61-1.12) | 34 | 0.49 (0.34-0.72) |
| **8** | ***68*** | 56 | 0.82 (0.60-1.13) | 53 | 0.78 (0.57-1.07) | 63 | 0.92 (0.68-1.25) |
| **9** | ***54*** | 46 | 0.85 (0.60-1.21) | 51 | 0.94 (0.67-1.31) | 52 | 0.96 (0.68-1.34) |
| **10** | ***41*** | 42 | 1.02 (0.70-1.48) | 38 | 0.92 (0.63-1.35) | 41 | 0.99 (0.67-1.45) |
| **11** | ***28*** | 32 | 1.17 (0.76-1.81) | 32 | 1.15 (0.75-1.78) | 42 | 1.52 (1.02-2.28) |
| **12** | ***26*** | 43 | 1.36 (0.93-2.01) | 38 | 1.21 (0.81-1.80) | 33 | 1.05 (0.68-1.61) |
| **<1 year** | ***41*** | **38** | **0.92 (0.83-1.03)** | **37** | **0.89 (0.79-1.00)** | **36** | **0.87 (0.77-0.97)** |

** per 100,000 children*

*2002-2006 timeframe was considered as a reference for the pre-vaccine baseline*
